# Does the Effect of Mental Fatigue Created by Motor Imagery on Upper Extremity Functions Change with Diaphragmatic Breathing Exercises? A Randomized, Controlled, Single-Blinded Trial

**DOI:** 10.3390/medicina60071069

**Published:** 2024-06-28

**Authors:** Ozan Bahadır Türkmen, Burçin Akçay, Canan Demir, Ahmet Kurtoğlu, Madawi H. Alotaibi, Safaa M. Elkholi

**Affiliations:** 1 Physical Therapy and Rehabilitation, Health Sciences, Bandirma Onyedi Eylul University, 10200 Balıkesir, Türkiye; 2 Department of Coaching Education, Faculty of Sport Science, Bandirma Onyedi Eylul University, 10200 Balıkesir, Türkiye; 3 Department of Rehabilitation Sciences, College of Health and Rehabilitation Sciences, Princess Nourah bint Abdulrahman University, P.O. Box 84428, Riyadh 11671, Saudi Arabia

**Keywords:** cognitive fatigue, upper extremity performance, muscle strength, sensation, hand dexterity

## Abstract

*Background and Objectives*: This study focused on the impact of mental fatigue induced by motor imagery on upper limb function, an area with limited research compared to lower limb performance. It aimed to explore how diaphragmatic breathing exercises influence these effects. *Materials and Methods*: This study included 30 participants, and Group 1 participated in 12 sessions of diaphragmatic breathing exercises under the supervision of a physiotherapist; Group 2 did not receive any intervention. For all the participants, mental fatigue was induced with motor imagery before and after the intervention, and evaluations were performed before and after mental fatigue. Upper extremity functions were evaluated using isometric elbow flexion strength, hand grip strength, upper extremity reaction time and endurance, finger reaction time, the nine-hole peg test, shoulder position sense, light touch-pressure threshold, and two-point discrimination. *Results*: The study results showed that after mental fatigue, there was a decrease in isometric elbow flexion strength, nondominant hand grip strength, and nondominant upper extremity endurance, and an increase in nondominant tactile sensation (*p* < 0.05). No changes were found in two-point discrimination, nine-hole peg test time, and position sense on either side (*p* > 0.05). The effect of mental fatigue on isometric elbow flexion strength and nondominant grip strength showed significant improvement following diaphragmatic breathing exercises (*p* < 0.05). *Conclusions*: This study found that mental fatigue from motor imagery can impact elbow flexion, hand grip strength, upper extremity endurance, and tactile sensitivity. Breathing exercises may help improve strength parameters affected by mental fatigue. It is crucial to consider these effects on upper extremity functions in rehabilitation programs.

## 1. Introduction

Mental fatigue is a psychobiological condition characterized by subjective feelings of tiredness and lack of energy caused by prolonged cognitive activities [1,2,3]. While mental fatigue has been widely observed to cause a decrease in cognitive performance, such as an increase in reaction time, an increase in the number of errors in simple mental tasks, and a decrease in attention [4,5,6], the effects on exercise performance have been found to be related to the type of physical task. According to recent reviews in the literature, mental fatigue has a negative effect on endurance, sports motor skills, and decision-making performances [2,7,8], while maximal strength, power, and anaerobic performance are generally not affected. High-intensity anaerobic exercises are likely to cause peripheral fatigue resulting from changes at or beyond the neuromuscular junction. These exercises typically involve expending all energy initially, leading to rapid fatigue and relying on non-oxygen-based metabolic pathways. High-intensity anaerobic exercises require fewer decision-making processes than endurance performance due to their shorter duration and all-out strategy. In this context, one reason specific performances, such as maximal strength, may be unaffected by mental fatigue is that these performances are short and involve expending all energy at the start [2].

Habay et al. [9] demonstrated the detrimental effect of mental fatigue on whole-body maximal dynamic endurance performance in a meta-analysis study [9]. The impact of mental fatigue on lower extremity performance and whole-body endurance has often been studied [7,8,9]. However, studies investigating the effects on upper extremity function are limited [10,11,12,13,14]. These studies have shown an overall negative impact on performance in upper limb sports such as cricket, table tennis, and marksmanship [12,13,14]. Duncan et al. [11] also reported that dexterity decreased after mental fatigue [11]. Valenza et al. [15] showed that mental fatigue decreased performance in simple manual tasks, and Rozand et al. [16] reported that tired volunteers were slower in the performance of a speed–accuracy marking task [15,16]. However, in those three studies, sample sizes were small, and only hand functions were examined with manual dexterity or speed–accuracy marking tasks. In contrast to those studies, Budini et al. [10] suggested that mental fatigue in healthy individuals does not affect the neuromuscular parameters showing strength stability and dexterity when measured under single-task conditions.

Breathing therapy, also known as “diaphragmatic breathing” or “deep breathing”, is defined as an effective, integrative method of body/mind training to deal with stress and psychosomatic conditions [17]. Previous studies have shown that diaphragmatic breathing can initiate relaxation responses in the body, and benefit physical and mental health [18]. Although breathing with yoga techniques has been reported to reduce visual and auditory reaction time [19,20], there is no study in the literature that has examined the effect of breathing techniques on the functional parameters of the upper extremity. There is a limited number of studies in the literature that have examined the effects of mental fatigue on upper extremity parameters, and there is no study that has examined the change in the effects of mental fatigue with diaphragmatic breathing exercises. Therefore, the aim of this study was to investigate the effects of mental fatigue on upper extremity performance (upper extremity endurance, elbow flexion and hand grip strength, position sense, upper extremity and finger reaction time, hand performance, light touch-pressure threshold, tactile sensation, and two-point discrimination) and to examine whether these effects change with diaphragmatic breathing exercises.

The negative effects of mental fatigue on lower extremity functions and endurance performance have been extensively studied. However, there are limited studies on its effects on upper extremity functions and how diaphragmatic breathing exercises might alter these effects. Therefore, the primary aim of our research is to investigate the effects of mental fatigue induced by motor imagery on upper extremity functions. Secondly, we aim to examine whether these effects change with diaphragmatic breathing exercises.

## 2. Materials and Methods

The study design was planned as a randomized, controlled, single-blinded study. Since it was thought that mental functions might be affected, all the procedures to be applied were explained to the participants, and written informed consent was obtained, but the purpose and hypotheses were not explained, as in a similar study [21]. After the final assessment of the participant was completed, the purpose and hypotheses of this study were explained. One of the researchers (C.D.) performed the diaphragmatic breathing exercises, and the other researchers (O.B.T. and B.A) who performed the evaluations were blinded to the subject groups, thereby ensuring that this study was single-blinded. The researchers (O.B.T. and B.A.) conducted the evaluations without knowing which group the participants were in. To avoid any bias, group information was kept confidential, and the statistical analyses were conducted by another researcher (A.K.).

Approval for this study was granted by the Non-Interventional Ethics Committee of Bandırma Onyedi Eylül University Faculty of Health Sciences (date number: 12 November 2021-2021-29, number: 2021-64). All procedures performed followed the 1964 Declaration of Helsinki and its later amendments or comparable ethical standards. Participants who wanted to participate in this study signed the informed consent form. Participants were assured that the data obtained would only be used for research purposes. The clinical trial number of this study is NCT05236244.

### 2.1. Participants

This study included students in the Department of Physiotherapy and Rehabilitation of Bandirma Onyedi Eylul University Faculty of Health Sciences. The participants were aged 18–30, with a sedentary lifestyle, who volunteered to participate in this study, and who did not have any problems that would prevent respiratory exercise. Individuals with any chronic disease, sensory problems, trauma in the last six months, or taking regular medication were excluded from this study.

The 30 participants in our study were randomly assigned to two groups (intervention group *n* = 15; control group *n* = 15) using a simple random number table. The demographic characteristics of the two groups were similar based on age, gender, and physical characteristics. To ensure the confidentiality of the assignment, the researchers (B.A. and O.B.T.) needed to know which group the participants were assigned to during the randomization process. The participants used in our study represent a homogenous group; 30 sedentary individuals of similar age, gender, and physical characteristics were studied. This may limit the generalization of the results to more extensive and diverse populations. Results may differ in individuals with different age groups, levels of physical activity, or various health conditions.

### 2.2. Assessments

Age, gender, weight, height, and inclusion and exclusion criteria were questioned using a demographic information form. Two participants were excluded due to a history of trauma, and one was excluded due to chronic disease.

The Edinburgh Hand Preference Questionnaire, proven valid and reliable in Turkish, was used to determine hand dominance [22]. 

All assessments were carried out in the laboratory over four days: two days for the first assessment and two days for the final evaluation four weeks later. Participants were taught the tests on the first day before all tests. After four weeks, participants were shown the tests again on the first assessment day. The intervention group performed diaphragmatic breathing exercises for four weeks, while the control group received no intervention. A one-week break was taken between the two different assessment days to eliminate the effect of mental fatigue. All assessments were conducted in the morning (8:30–10:00) to minimize the effect of mental fatigue. All participants were asked to sleep at least 7 h on the night before the assessment and to refrain from alcohol consumption and vigorous physical activity the previous day. Participants were also asked not to consume caffeine and nicotine at least 3 h before the test and to report whether they were taking any medication or had any acute illness, injury, or infection [1]. 

### 2.3. Upper Extremity Performance Tests 

Isometric elbow flexion strength was measured with a Lafayette Hand-Held Dynamometer (Lafayette Instrument Company, Lafayette, IN, USA). When instructed, the participant pulled the hand towards the device, trying to move it as forcefully as possible towards the shoulder for 2–3 s. During that time, the examiner gave verbal encouragement. The examiner ensured no compensatory movements in trunk extension or lateral flexion away from the tested arm. The best of the three trials (measured in kilograms) was recorded as the participant’s test score. Thirty seconds of rest between successive trials controlled for fatigue [23]. 

Hand grip strength was measured with a Jamar^®^ Hydraulic Hand Dynamometer. The standard test position of the American Association of Hand Therapists was used. The participant was asked to grasp the dynamometer as strongly as possible and release it completely relaxed. Measurements were taken three times for both hands, and the mean values were recorded.

Upper extremity reaction time was measured using the validated Blazepod Trainer device (Blazepod Trainer Device, Play Coyotta Ltd., Tel Aviv, Israel) [24]. An evaluation panel consisting of 4 sensors was used for measurement. The flashing of the lights was automatically randomized by the device, and each test set was set to include ten flashes of the sensors through the blazed program. The subjects were asked to touch the illuminated sensor on the panel with their hands as quickly as possible. The time between the light appearing and the subject touching and extinguishing the illuminated sensors was recorded by the device in milliseconds for both hands separately [25]. 

Finger reaction time was evaluated with the Human Benchmark test, one of the computer game tests [26]. The subject was asked to press the button when the red box turned green on the computer screen. Five practice trials were performed for each hand, followed by ten experimental trials. The time between the light stimulus and the subject’s response was recorded in milliseconds, and the test was repeated for both hands [27]. 

The 9-hole peg test (9-DPT) was used to assess hand performance. The test was repeated for both hands. The subjects were instructed to insert the pegs into the holes and then remove the pegs from the nine holes in the shortest time possible. Removal and insertion times were recorded in seconds as a total time [28]. 

The position sense of the shoulder joint was measured with a digital goniometer (Baseline^®^, Evaluation Instruments, Nailsea, UK). The measurement was performed in a quiet, calm environment in an isolated room with a closed door. The eyes of the subject were closed to eliminate visual stimuli. The shoulder joint movements in the target angles were actively taught three times, and the subject was asked to learn this position by holding it for 3 s. The subject was positioned standing with feet shoulder-width apart and was then instructed to raise the arm from a position adjacent to the body to the angles marked as the target points of 55°, 90°, and 125°. During the test, the subject actively moved the arm to the target angle, and the absolute deviation from the target angle was recorded. The testing order was randomized, each test was repeated three times, and the average value was recorded. The test was performed on both sides. A rest period of 10 s was given between repetitions and 1 min between tests [29]. 

The light touch-pressure threshold was evaluated with the Semmes Weinstein monofilament test (North Coast Medical, Morgan Hill, CA, USA) [30]. Filaments were progressively applied to the palmar surface of the index fingers of the subject, who was instructed to keep their eyes closed [31]. To progress, the subject was required to correctly identify two of the three stimuli presented at each level [27]. The test score was recorded as the correctly identified calibrated force (in grams) of the smallest filament.

The two-point discrimination test was performed with an esthesiometer (Baseline^®^ White Plains, New York, NY, USA). The participants were seated with the palmar side of the hand pointing upwards, elbow flexed 90 degrees, and eyes closed. Randomly, one or two prongs were touched over the hand, with as little pressure as possible, and the individual was asked whether one or two prongs were felt. The smallest inter-prong distance for which the subject gave two correct responses out of three trials was recorded in millimeters for that site. The shortest distance range the participant could feel was recorded in millimeters (mm) [30]. 

In the upper extremity endurance test, after weighing the body weight, the weight to be held was calculated as 2% of the body weight. The subject lay face down on the bed with the arm to perform the test hanging free. The subject was then asked to lift the weight with the thumb pointing up and the arm at 90° in the horizontal plane. A metronome set to 60 Hz was then used to standardize the test, whereby the subject was instructed to raise their arm on the first beat, hold it in 90° abduction for the next beat, and then lower it to the starting position on the third beat. The test was terminated when any of the following termination criteria were met [32]:

(1) Inability to hold/maintain the arm at 90° in the horizontal plane.
(2) Failure of the metronome to maintain its rhythm.
(3) Compensatory movement made by the scapula.
(4) Inability of the glenohumeral joint to maintain lateral rotation position.
(5) Use of trunk rotation to achieve the required range/position.
(6) Use of lower limbs to provide lifting.
(7) Raising the other arm.
(8) The subject stated that they could not continue [32].

### 2.4. Interventions

Mental fatigue was created with motor imagery. The specific parameters used in motor imagery are based on certain reasons. The hypothesis that mental fatigue may arise due to the involvement of sustained attention and inhibition processes during motor imagery has led researchers to demonstrate that prolonged motor imagery can induce mental fatigue. For example, Rozand et al. (2016) [33] showed that 100 imagined contractions (approximately 30 min) lead to mental fatigue and increase movement duration during a pointing task. The optimal duration for motor imagery has been reported to be around 17 min, but positive effects of motor imagery seem to disappear when this duration is exceeded. Therefore, the parameters used in our study were selected based on these durations and mechanisms identified in previous research. For motor imagery, the subjects were first taught isometric biceps brachii contractions and were then instructed to perform 150 submaximal isometric biceps contractions for the exercise at 50% of 1 maximum voluntary contraction (MVC). If the physical exercise was too difficult during the training session (i.e., excessive shoulder and trunk movements, signs of sweating), the target force was fixed at 45% of the MVC. If the physical exercise was very easy (i.e., low perceived exertion and no muscle fatigue at the end), the target force was fixed at 55% of the MVC. At the end of the exercise, the subject performed maximal imaginary isometric contractions of the right elbow flexors (biceps muscle) for motor imagery. To induce mental fatigue with motor imagery (MI), the subject was instructed to perform an imaginary elbow flexion while not contracting the muscle in any way. The subjects performed four blocks (duration of one block = 12.5 min; total prolonged MI duration = 53 min), with 50 imaginary contractions, each imaginary contraction lasting 5 secs followed by a rest period of 10 secs and a rest period of 1 min between blocks. Three instructions were given during prolonged MI. Two seconds before initiating the imaginary contraction, participants heard “Get ready”, followed by “Pull” to initiate the imaginary contraction and “Relax” to stop the imaginary contraction. Whether the subject contracted the biceps muscle during MI was checked with an EMG-Biofeedback device (NeuroTrac^®^ MyoPlus4 Pro, Verity Medical, Romsey, UK) with the researcher sitting 1 m behind the subject [21]. 

**Figure 1:**
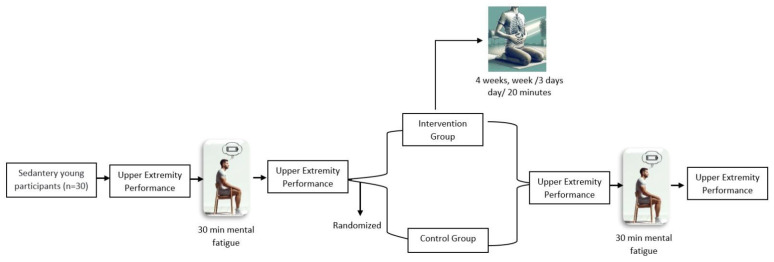
Experimental Design of Study.

Diaphragmatic breathing exercises were performed for the respiratory group for 4 weeks, 3 days a week, 20 min a day, providing a total of 12 sessions. The sessions were conducted by the same professional physiotherapist (C.D.). During the first five and last five minutes of the session, the subjects were instructed to focus on their breathing and the sensations produced in the body while sitting comfortably in a chair with all joints relaxed and the eyes closed. After the initial 5 min of relaxed breathing, the subjects were instructed to perform diaphragmatic breathing for 10 min by placing their dominant hand on their abdomen and the other hand on their chest, contracting their diaphragm and inflating their abdomen while inhaling deeply through the nose, relaxing their diaphragm muscles during the exhalation phase and drawing their abdomen in while slowly exhaling through the mouth. The subjects were asked to perform six diaphragmatic respirations per minute. After 10 min of diaphragmatic breathing exercises, the session was terminated after 5 min of relaxed breathing, as at the beginning [34] (Figure 1).

### 2.5. Sample Size

The sample size was determined based on a previous study investigating the physical effects of mental fatigue induced by motor imagery [21]. Accordingly, the sample size of the study was calculated as at least 15 individuals in each group (considering dropouts) with a 95% confidence interval and 80% power by G*Power 3.1.9.7 analysis.

### 2.6. Statistical Analysis

Statistical analysis was performed using IBM SPSS Statistics software for Windows, ver. 23.0 (IBM Corpn., Armonk, NY, USA). Conformity of the data to normal distribution was assessed using the Shapiro–Wilk test. Descriptive statistics for measured variables were given as mean ± SD and minimum/maximum values. To evaluate the effect of mental fatigue, the Wilcoxon paired two-sample test was used to compare the values before and after mental fatigue. To compare the dependent variables before and after mental fatigue within the group, the one-sample *t*-test was used for data with normal distribution, and the Wilcoxon paired two-sample test analysis was used for data not conforming to normal distribution. In intergroup comparisons, the independent samples *t*-test was applied to the delta value obtained by taking the difference between the values before and after mental fatigue for those that fit normal distribution and the Mann–Whitney *U* Test for those that did not. The level of statistical significance was accepted as *p* < 0.05 for all tests.

## 3. Results

The demographic characteristics of both groups were determined to be similar (*p* > 0.05) (Table 1). The comparisons of the values of all the subjects before and after mental fatigue are shown in Table 2. Following the mental fatigue protocol, a statistically significant decrease was determined in isometric elbow flexion strength, nondominant hand grip strength, and nondominant upper extremity endurance, as well as an increase in nondominant tactile sensation and dominant finger-press reaction time (*p* < 0.05). No statistically significant change was found in upper extremity reaction time, nine-hole peg test duration, two-point discrimination, or position sense on either side (*p* > 0.05) (Table 2). The values of the study group and control group before and after mental fatigue at baseline and after four weeks are given in Table 3. To evaluate the effect of mental fatigue, delta values were subtracted by taking the difference between before and after mental fatigue. When the baseline and post-treatment delta values were analyzed, only the change in isometric elbow flexion strength on the dominant and nondominant side and nondominant grip strength showed a statistically significant decrease with mental fatigue in the respiratory exercise group (*p* < 0.05). In the control group, a statistically significant decrease was observed in the change in isometric elbow flexion strength on the dominant and nondominant side with mental fatigue (*p* < 0.05). There was a significant difference between the two groups only in the amount of change in dominant hand isometric elbow flexion strength in mental fatigue after the intervention (*p* < 0.05) but not in all other parameters (*p* > 0.05) (Table 4).

## 4. Discussion

The results of this study demonstrated a significant decrease in isometric elbow flexion strength, nondominant hand grip strength, and nondominant upper extremity endurance, along with an increase in nondominant tactile sensation following mental fatigue. No statistically significant changes were observed in two-point discrimination, nine-hole peg test duration, or position sense on either side. These findings align with previous research indicating that mental fatigue adversely affects specific aspects of physical performance, particularly those involving endurance and complex motor tasks [7,8]. When comparing the intervention and control groups, it was observed that diaphragmatic breathing exercises significantly mitigated the decrease in isometric elbow flexion strength and nondominant grip strength induced by mental fatigue. This suggests that diaphragmatic breathing may serve as an effective intervention to counteract the adverse effects of mental fatigue on muscle strength. These results consistently show that breathing exercises can activate parasympathetic tone, improve neural processing speed, and enhance physical performance parameters [17,18].

Mental fatigue is traditionally induced by prolonged tasks such as the AX-Continuous Performance Task (AX-CPT) [1,35] or the Stroop task [16]. Motor imagery, which includes sustained attention and inhibitory processes, can also lead to mental fatigue, with 100 imaginary contractions shown to cause it [21,33]. Motor imagery is the internal simulation of movement without actual motor output and is divided into visual and kinesthetic types [36]. This study used motor imagery, which involves imagining the sensations produced by the task and activates brain regions like the premotor cortex, parietal cortex, and cerebellum. The protocol of Jacquet et al. [21] was used to induce mental fatigue with motor imagery. Studies examining the effects of mental fatigue have focused on endurance performance, but studies on the effects of mental fatigue on strength are limited [7,8,9,37,38]. In a study by Nakashima et al. [37], it was observed that after mental fatigue induced by the motor imagery of lifting a 500 mL and 1500 mL water-filled bottle with both hands, a decrease in pinch grip strength was determined only after the imagery of lifting a 1500 mL water-filled bottle. Nakashima et al. [38] also reported a significant decrease in pinch grip strength after mental fatigue induced by a visual-motor task at 50% of maximum right-hand pinch grip strength. In the current study, mental fatigue was induced by motor imagery of 50% of the maximum elbow isometric force, similar to the method used in previous studies [21,38], and mental fatigue was found to be associated with decreased bilateral isometric elbow strength and nondominant hand grip strength. Regarding the decrease in muscle strength, Nakashima et al. [38] stated that mental fatigue occurs with the constant repetition of motor imagery and that mental fatigue can affect performance recovery in the same way as physical fatigue. Mental fatigue induced by motor imagery is also to thought to affect muscle strength by reducing corticospinal excitability in the central nervous system. While both hands were used in one of the studies by Nakashima et al. [37], only the right hand was used in the other study. In the current study, the dominant and nondominant hands were evaluated separately [37]. In particular, hand grip strength was seen to be reduced on the nondominant side despite the dominant isometric elbow flexion imagery. The mechanism underlying the difference in mental fatigue between the dominant and nondominant sides should be investigated in future studies.

In a meta-analysis and systematic review examining the negative effects on endurance of mental fatigue, the endurance addressed in those studies was lower extremity endurance performance and whole-body endurance [8,9]. Although studies on upper extremity endurance parameter changes after mental fatigue are limited in the literature, Dallaway et al. [39] stated that rhythmic handgrip endurance performance decreases. In the current study, similar to the other studies, after the mental fatigue induced by motor imagery, there was a decrease in endurance on both sides, but only nondominant upper extremity endurance was determined to have statistically decreased. The effect of mental fatigue on endurance performance has been explained as the effect on neurotransmitter systems in more than one brain region, and the sum of these effects may lead to deterioration in endurance performance [8,40]. Changes in neurotransmitter systems can directly influence observed performance metrics by altering the brain’s ability to manage effort and fatigue. For instance, a noradrenaline reuptake inhibitor decreases endurance performance by increasing the perceived effort without affecting intramuscular fatigue. In contrast, a dopamine reuptake inhibitor can enhance performance by allowing for higher power output without changing perceived exertion levels. These findings suggest that multiple neurotransmitter systems across different brain regions contribute to the effects of mental fatigue on endurance performance, with each system playing a role in regulating perceived effort and motivation [8]. In addition, when more motor units are activated, and the muscle mass involved in contraction increases, both central and peripheral drives can be greater. Since the number of muscle fibers in the motor unit is lower in the arms than in the legs and the number of motor neurons activated to produce force differs [41], it can be recommended that further studies should examine the differences in the effects of mental fatigue in the lower and upper limbs. 

Reaction time, which is an important factor for performance, is defined as the time between a stimulus and the reaction to that stimulus. A systematic review by Habay et al. [7] showed that sport-specific psychomotor reaction time was negatively affected by mental fatigue. Migliaccio et al. [42] observed increased finger reaction with the dominant hand after mental fatigue induced by the Stroop test but the test was not performed on the nondominant hand. In the present study, reaction tests were performed with both hands, and hand-finger press reaction time increased after mental fatigue, similar to the findings of previous studies [7,42], but upper limb reaction time was not affected. Since smaller muscle groups (small motor units) are more active in the finger reaction test than in the upper extremity reaction test, small muscle groups may have been more affected by mental fatigue. However, the reason that the nondominant hand was less affected needs to be investigated in future studies.

When the effect of mental fatigue on manual dexterity was examined with the Minnesota Manual Dexterity Rotation Test in a study by Duncan et al. [11], it was stated that manual dexterity was negatively affected. In contrast, hand dexterity was examined with a nine-hole peg test in the current study, and no effect of mental fatigue on dexterity was observed.

Proprioception includes two components, the position sense of the limbs and the sense of limb movement (kinesthesia). In this study, limb position sense was evaluated, and it was observed that mental fatigue induced by motor imagery had no negative effect on shoulder position sense. No previous study on the effect of mental fatigue on shoulder position sense could be found in the literature. In a meta-analysis by Takasaki et al. of shoulder position sense, it was stated that physical fatigue of the shoulder muscles affected shoulder external rotation position sense but not other shoulder movements [43]. In this study, position sense with shoulder flexion was evaluated, and the use of external rotation of the shoulder may have changed the results. 

Static two-point discrimination and Semmes Weinstein monofilament tests are the most commonly used tests for the evaluation of sensory impairment and can also be used in healthy individuals [30]. In the current study, mental fatigue induced by motor imagery was determined to produce light touch-pressure sensation impairment, which was statistically significant in the nondominant hand but still within the normal sensory threshold limits. However, static two-point discrimination did not change after mental fatigue. The light touch-pressure sensation is perceived by superficial receptors and transmitted to the sensory cortex, whereas static two-point discrimination is a more complicated sensation that is perceived by deep and superficial receptors and integrated with inhibitory mechanisms in the central nervous system. Tamura et al. [44] stated that static two-point discrimination is a sensation processed by peripheral factors and central factors involved in the comparison process between two-point stimuli. Therefore, the results of this study were not anticipated. The effects of mental fatigue on motor responses have been extensively studied, but in the current study, the effect on sensory components was examined for the first time. It can be recommended that further studies examine the effects on the peripheral and central sensory structures.

Although most of the studies on the effects of diaphragmatic breathing exercises on physical performance and skills consist of studies on conditions in which the balance of the autonomic nervous system is disrupted, such as sleep disorders, anxiety, and depression, very few studies have investigated the effects of breathing exercises on physical performance parameters in healthy individuals [18,19]. Previous studies have reported that parasympathetic tone is activated by breathing exercises, neural processing speed is increased, and positive improvements are obtained in physical performance parameters [18,45]. In the current study, the effect of mental fatigue on isometric elbow flexion strength and nondominant grip strength showed significant improvement with diaphragmatic breathing exercises, but no superiority of this change over the control group was observed. Similar to the findings in the literature, these results suggest that respiratory exercises can be used as an effective method to reduce the effects of mental fatigue on physical performance parameters, including muscle strength [45]. In future studies, the effects of different respiratory exercise protocols on mental fatigue and upper extremity performance can be investigated to provide a clear methodology and increase the level of evidence.

### Limitations and Further Research

The main limitation of this study was that the severity of mental fatigue induced by motor imagery was not measured, as the severity of fatigue felt may be perceived differently from one person to another. Another limitation can be considered to be that the study sample comprised only young adults, and the effects of mental fatigue may be different in different age groups. Although the number of male and female subjects was not equal, the demographic characteristics were similar in both groups. The gender imbalance may not constitute a limitation as it has been reported that personal characteristics such as gender, age, BMI, and physical fitness level do not affect susceptibility to mental fatigue [9]. A questionnaire about quality of life was not used in this study, but it can be used in future studies. The strength of this study was that it is the first study in the literature to have examined the effects of mental fatigue on upper extremity performance tests (endurance, reaction time, isometric elbow flexion, light touch, two-point discrimination, and position sense) and investigate whether respiratory exercises are effective in reducing these effects. Thus, it can be considered of value as a pioneering study to determine the effects of mental fatigue on the upper extremities of athletes and neurological disease patient groups as well as healthy individuals. In future research, it is necessary to examine the effects of mental fatigue on peripheral and central sensory structures in more detail. This is important to better understand the effects of mental fatigue on sensory components. Furthermore, different respiratory exercise protocols must be examined to determine the effects on mental fatigue and upper limb performance. This is a critical step in establishing a transparent methodology and increasing the level of evidence. It is also essential to corroborate the effects of mental imagery-induced mental fatigue on upper limb parameters with more objective methods, such as electroencephalography. These approaches may help to identify interventions to reduce the effects of mental fatigue and are valuable for considering the effects of mental fatigue on upper limb function in rehabilitation programs. Such studies may provide valuable information for athletes, patient groups with neurological diseases, and healthy individuals.

## 5. Conclusions

The results of the current study indicated that mental fatigue caused by motor imagery may affect elbow flexion and hand grip strength, upper extremity endurance, and tactile sensitivity, and the effect on strength parameters can be significantly improved with diaphragmatic breathing exercises. There is a need for future studies to examine the effects of mental fatigue on the upper extremities and interventions to reduce these effects and for the impact of mental fatigue induced by motor imagery on upper extremity functions to be considered in the rehabilitation program.

## Figures and Tables

**Table 1:** Demographic characteristics of the participants.

	Total (*n*: 30)	Intervention Group (*n*: 15)	Control Group (*n*: 15)	*p* Values
Age (years)	21.10 ± 1. 17 (20–29)	21.20 ± 2.27 (20–29)	21.00 ± 0.92 (20–23)	0.500
Height (cm)	168.90 ± 8.96 (155–188)	167.86 ± 7.32 (155–184)	169.93 ± 10.49 (155–188)	0.755
Weight (kg)	63.52 ± 12.87 (44–97)	61.20 ± 10.79 (48–83)	65.83 ± 14.67 (44–97)	0.340
BMI (kg/cm^2^)	22.12 ± 3.24 (17.00–30.10)	28.70 ± 3.33 (17.00–28.70)	22.56 ± 3.18 (17.90–30.10)	0.384

cm: centimeter, kg: kilogram, BMI: Body Mass Index, Mann-Whitney *U* Test was used for *p*-value.

**Table 2:** Comparison of pre and post-fatigue values of all participants.

*n* = 30	Pre-Fatigue	Post-Fatigue	*p*
Isometric elbow flexion strength-D (kg)	23.35 ± 7.68 (14.10–43.00)	19.25 ± 6.70 (12.40–40.30)	0.001
Isometric elbow flexion strength-ND (kg)	23.45 ± 7.72 (16.20–42.10)	19.50 ± 6.91 (12.80–38.80)	0.000
Handgrip strength-D (kg)	28.00 ± 9.53 (20.00–55.00)	26.00 ± 10.56 (18–53)	0.173
Handgrip strength-ND (kg)	28.00 ± 10.11 (18.00–55.00)	23.00 ± 10.58 (16.00–54.00)	0.008
Upper extremity reaction time-D (ms)	662.00 ± 74.17 (532.00–797.00)	642.50 ± 93.66 (459–820)	0.805
Upper extremity reaction time-ND (ms)	660.00 ± 90.65 (484–817)	631.50 ± 103.61 (489–898)	0.910
Finger-press reaction time-D (ms)	247.00 ± 36.06 (168–335)	262.50 ± 40.90 (197–365)	0.033
Finger-press reaction time-ND (ms)	251.50 ± 33.67 (201–329)	256.00 ± 36.28 (205–374)	0.371
9-Hole peg-D	15.89 ± 1.94 (13.24–21.98)	15.13 ± 1.91 (11.27–20.53)	0.070
9-Hole peg-ND	17.43 ± 2.45 (14.47–24.53)	16.78 ± 1.80 (13.21–20.61)	0.050
Endurance-D (ms)	110.03 ± 55.48 (55.50–290.60)	89.77 ± 72.89 (53.90–345.15)	0.349
Endurance-ND(ms)	100.67 ± 55.64 (50.00–305.77)	90.14 ± 58.62 (56.00–303.12)	0.041
Tactile sensitivity-D (g)	2.36 ± 0.26 (1.65–2.83)	2.44 ± 0.21 (1.65–2.83)	0.083
Tactile sensitivity-ND (g)	2.36 ± 0.30 (1.65–2.83)	2.44 ± 0.24 (1.65–2.83)	0.009
Two-point discrimination-D (mm)	3.00 ± 0.90 (1.00–4.00)	3.00 ± 0.94(1.00–4.00)	0.837
Two-point discrimination-ND (mm)	2.00 ± 0.73 (1.00–4.00)	3.00 ± 0.72(1.00–4.00)	0.132
Position sense 125°-D	6.00 ± 3.02 (1.65–14.45)	5.07 ± 3.81 (0.80–15.85)	0.213
Position sense 125°-ND	5.87 ± 3.81 (1.80–15.30)	6.10 ± 3.79 (2.05–16.25)	0.713
Position sense 90°-D	3.02 ± 1.81 (0.80–7.25)	3.07 ± 1.76 (0.75–7.05)	0.931
Position sense 90°-ND	3.10 ± 1.74 (0.80–7.55)	3.42 ± 2.19 (1.35–10.80)	0.504
Position sense 55°-D	5.57 ± 3.58 (1.20–12.15)	5.25 ± 3.65 (1.25–14.80)	0.992
Position sense 55°-ND	5.15 ± 3.33 (1.45–12.35)	4.87 ± 3.39 (0.95–15.40)	0.213

mm: millimeter; ms: millisecond; °: degree; D: dominant; ND: nondominant. Wilcoxon signed rank test was used for *p*-value.

**Table 3:** Pre- and post-mental fatigue results of the groups at baseline and after four weeks.

		Intervention		Control	
		Baseline	4 Weeks Later	Baseline	4 Weeks Later
Isometric elbow flexion strength-D (kg)	PrF PsF	27.34 ± 6.32 (16.40–38.60) 21.33 ± 4.09 (15.20–29.10)	19.21 ± 6.77 (13.00–36.00) 18.19 ± 5.62 (10.90–32.60)	24.33 ± 8.79 (14.10–43.00) 22.46 ± 8.69 (12.40–40.30)	19.25 ± 7.61 (11.80–34.90) 20.81 ± 7.85 (10.40–34.60)
Isometric elbow flexion strength-ND (kg)	PrF PsF	26.54 ± 5.50 (18.30–38.80) 20.76 ± 3.38 (13.90–25.40)	19.47 ± 5.97 (14.30–34.00) 18.78 ± 6.02 (11.20–32.70)	25.05 ± 9.59 (16.20–42.10) 21.65 ± 9.32 (12.80–38.80)	19.80 ± 7.75 (11.80–34.10) 20.50 ± 8.74 (11.10–40.20)
Handgrip strength-D (kg)	PrF PsF	29.33 ± 6.70 (21.00–48.00) 27.93 ± 6.31 (21.00–44.00)	29.00 ± 6.19 (22.00–48.00) 28.33 ± 8.63 (18.00–48.00))	31.53 ± 11.85 (20.00–55.00) 31.23 ± 13.60 (18.00–53.00)	31.33 ± 12.92 (18.00–56.00) 31.45 ± 13.99 (15.00–56.00)
Handgrip strength-ND (kg)	PrF PsF	28.33 ± 6.88 (20.00–48.00) 24.20 ± 6.83 (16.00–42.00)	26.33 ± 6.27 (19.00–45.00) 26.86 ± 8.30 (17.00–49.00)	30.66 ± 12.70 (18.00–55.00) 29.20 ± 13.10 (16.00–54.00)	31.46 ± 14.07 (19.00–58.00) 30.62 ± 13.43 (16.60–57.00)
Upper extremity reaction time-D (ms)	PrF PsF	669.26 ± 74.77 (544–797) 675.33 ± 83.67 (566–820)	580.33 ± 97.14 (455–806) 586.33 ± 90.23 (414–775)	638.20 ± 72.72 (532–760) 618.00 ± 97.00 (459–752)	602.20 ± 111.14 (441–779) 581.04 ± 82.18 (165–761)
Upper extremity reaction time-ND (ms)	PrF PsF	677.06 ± 74.77 (484–817) 674.20 ± 118.30 (490–898)	634.13 ± 109.07 (473–827) 638.66 ± 109.92 (408–787)	630.46 ± 63.58 (530–770) 619.66 ± 81.54 (489–831)	650.60 ± 103.38 (446–797) 608.50 ± 92.95 (459–770)
Finger-press reaction time-D (ms)	PrF PsF	244.93 ± 38.94 (168–335) 258.00 ± 26.83 (207–295)	256.26 ± 32.97 (216–323) 264.93 ± 53.91 (198–375)	252.06 ± 33.91 (195–317) 279.86 ± 49.89 (197–365)	266.33 ± 46.52 (177–357) 268.92 ± 33.93 (210–336)
Finger-press reaction time-ND (ms)	PrF PsF	251.20 ± 35.30 (201–329) 262.73 ± 40.29 (205–374)	256.40 ± 28.93 (206–319) 274.66 ± 68.71 (208–459)	262.73 ± 32.09 (216–323) 258.40 ± 33.06 (214–316)	−245.60 ± 35.69 (187–324) 259.54 ± 23.00 (210–336)
Endurance-D(ms)	PrF PsF	112.05 ± 35.92 (55.50–176.00) 127.23 ± 74.90 (82.22–329.49)	119.18 ± 51.50 (61.53–270.00) 103.39 ± 47.76 (45.14–241.23)	133.94 ± 69.48 (67.00–290.60) 11.38 ± 72.52 (53.90–345.15)	129.30 ± 69.01 (61.20–302.03) 121.03 ± 89.28 (50.90–428.00)
Endurance-ND (ms)	PrF PsF	103.04 ± 27.83 (72.69–173.94) 106.36 ± 59.22 (67.40–303.12)	92.58 ± 27.01 (65.00–168.40) 97.99 ± 36.42 (57.00–189.74)	134.05 ± 71.57 (50.00–305.77) 108.71 ± 60.07 (53.90–345.15)	102.58 ± 53.27 (55.43–264.40) 119.35 ± 95.78 (43.18–423)
Tactile sensitivity-D (g)	PrF PsF	2.32 ± 0.39 (1.65–2.83) 2.44 ± 0.28 (1.65–2.83)	2.49 ± 0.22 (2.36–3.22) 2.62 ± 0.34 (2.36–3.22)	2.36 ± 0.32 (1.65–2.83) 2.44 ± 0.11 (2.36–2.83)	2.43 ± 0.40 (1.65–3.22) 2.52 ± 0.28 (2.36–3.22)
Tactile sensitivity-ND (g)	PrF PsF	2.21 ± 0.37 (1.65–2.83) 2.39 ± 0.23 (1.65–2.83)	2.54 ± 0.25 (2.36–3.22) 2.62 ± 0.34 (2.36–3.22)	2.33 ± 0.19 (1.65–2.44) 2.41 ± 0.26 (1.65–2.83)	2.43 ± 0.40 (1.65–3.22) 2.54 ± 0.29 (2.36–3.22)
Two-point discrimination-D (mm)	PrF PsF	2.40 ± 0.98 (1–4) 2.26 ± 0.88 (1–4)	1.93 ± 0.73 (1–3) 2.00 ± 0.65 (1–3)	2.46 ± 0.83 (1–4) 2.66 ± 0.97 (1–4)	2.13 ± 0.74 (1–4) 2.06 ± 0.45 (1–3)
Two-point discrimination-ND (mm)	PrF PsF	2.33 ± 0.48 (2–3) 2.60 ± 0.50 (2–3)	2.13 ± 0.74 (1–3) 2.26 ± 0.59 (1–3)	2.60 ± 0.91 (1–4) 2.66 ± 0.89 (1–4)	2.20 ± 0.77 (1–4) 2.07 ± 0.70 (1–4)
Position sense 125°-D	PrF PsF	6.40 ± 3.25 (2.25–14.45) 6.07 ± 3.42 (2.50–15.85)	5.98 ± 3.16 (1.90–13.95) 5.80 ± 3.23 (2.35–14.05)	6.96 ± 2.85 (1.65–11.65) 6.00 ± 4.28 (0.80–14.35)	5.62 ± 4.01 (0.70–15.30) 5.04 ± 2.95 (1.90–12.90)
Position sense 125°-ND	PrF PsF	7.01 ± 4.09 (2.85–15.30) 7.06 ± 3.35 (2.05–13.40)	5.40 ± 2.73 (2.15–12.85) 5.40 ± 2.02 (2.20–10.75)	6.68 ± 3.63 (1.80–13.85) 6.49 ± 4.27 (2.15–16.25)	5.50 ± 3.17 (1.60–12.60) 5.52 ± 4.38 (1.60–18.80)
Position sense 90°-D	PrF PsF	3.30 ± 1.91 (0.95–6.90) 3.33 ± 1.69 (0.75–6.60)	3.58 ± 3.00 (0.95–11.95) 2.60 ± 1.04 (0.65–4.30)	3.61 ± 1.76 (0.80–7.25) 3.51 ± 1.87 (0.80–7.05)	3.13 ± 2.23 (0.50–8.15) 3.39 ± 2.85 (0.35–11.35)
Position sense 90°-ND	PrF PsF	3.68 ± 1.65 (1.60–7.55) 4.27 ± 2.06 (1.35–8.75)	3.57 ± 1.92 (1.40–8.80) 3.28 ± 2.37 (0.50–8.25)	3.10 ± 1.82 (0.80–7.10) 3.51 ± 2.30 (1.45–10.80)	3.03 ± 2.20 (0.50–8.70) 2.79 ± 1.42 (1.35–5.90))
Position sense 55°-D	PrF PsF	5.52 ± 3.85 (1.20–12.15) 6.01 ± 2.67 (1.85–11.00)	5.15 ± 2.79 (1.40–8.95) 5.34 ± 3.23 (2.05–14.30)	7.01 ± 3.23 (1.90–11.75) 6.18 ± 4.51 (1.25–14.80)	5.73 ± 3.68 (2.10–15.30) 4.60 ± 2.39 (0.85–9.10)
Position sense 55°-ND	PrF PsF	6.63 ± 3.34 (3.00–12.35) 5.13 ± 2.62 (1.40–9.15)	7.39 ± 4.75 (2.00–20.10) 6.68 ± 3.69 (1.75–13.85)	6.35 ± 3.41 (1.45–11.85) 5.82 ± 4.08 (0.95–15.40)	5.23 ± 3.46 (1.60–13.75) 4.16 ± 3.69 (0.65–15.60)
9-Hole peg-D	PrF PsF	16.40 ± 1.64 (13.24–19.63) 15.45 ± 2.33 (11.27–20.53)	14.30 ± 2.15 (10.71–17.54) 14.58 ± 2.09 (11.22–18.28)	15.75 ± 2.22 (13.39–21.98) 14.83 ± 1.40 (11.57–17.40)	14.44 ± 1.37 (11.95–16.47) 14.63 ± 1.82 (11.88–18.08)
9-Hole peg-ND	PrF PsF	17.59 ± 1.78 (14.9–22.11) 16.91 ± 1.66 (13.9–19.86)	16.31 ± 1.95 (12.32–19.20) 15.47 ± 1.39 (13.50–1749)	18.06 ± 3.02 (14.47–24.53) 16.82 ± 1.99 (13.21–20.65)	16.26 ± 2.99 (10.22–20.60) 16.15 ± 2.05 (12.98–20.72)

mm: millimeter; ms: milliseconds; g: gram; °: degree; D: dominant; ND: nondominant; PrF: pre-fatigue; PsF: post-fatigue.

**Table 4:** Results of the differences in the amount of change before and after the application of mental fatigue according to the groups.

	Time	Intervention Group (n:15)	*p* Values	Control Group (n:15)	*p* Values	*p* Values
Isometric elbow flexion strength-D(kg)	1 2	−6.00 ± 6.67 (−23.40–1.10) −1.02 ± 2.80 (−10.20–1.70)	0.008 ^b^	−1.87 ± 4.89 (−12.90–8.80) 1.56 ± 2.62 (−1.40–7.70)	0.033 ^a^	0.093 ^d^ 0.018 ^d^
Isometric elbow flexion strength-ND (kg)	1 2	−5.78 ± 4.23 (−15.30–0.70) −0.68 ± 1.56 (−3.40–1.60)	0.000 ^a^	−3.40 ± 4.07 (−13.00–5.80) 0.70 ± 4.76 (−2.90–13.90)	0.006 ^b^	0.065 ^d^ 0.983 ^d^
Handgrip strength-D (kg)	1 2	−1.40 ± 3.68 (−10.00–4.00) −0.66 ± 5.09 (−8.00–12.00)	0.687 ^a^	−0.30 ± 5.81 (−8.00–13.00) −0.87 ± 4.76 (−10.00–7.00)	0.695 ^a^	0.541 ^c^ 0.909 ^c^
Handgrip strength-ND (kg)	1 2	−4.13 ± 4.64 (−13.00–3.00) 0.53 ± 3.41 (−9.00–6.00)	0.001 ^b^	−1.46 ± 6.09 (−12.00–10.00) −0.84 ± 2.73 (−5.40–4.74)	0.684 ^a^	0.519 ^d^ 0.143 ^d^
Upper extremity reaction time-D (ms)	1 2	6.06 ± 95.38 (−179.00–174.00) 6.00 ± 82.28 (−146–164)	0.998 ^a^	−20.20 ± 123.25 (−234.00–144.00) −21.15 ± 108.72 (−245–182)	0.982 ^a^	0.843 ^c^ 0.447 ^c^
Upper extremity reaction time-ND (ms)	1 2	−2.86 ± 131.93 (−327.00–164.00) 4.53 ± 129.13 (−201–208)	0.882 ^a^	−10.80 ± 79.05 (−138.00–103.00) −42.09 ± 88.12 (−185–109)	0.312 ^a^	0.409 ^c^ 0.258 ^c^
Finger-press reaction time-D (ms)	1 2	13.06 ± 40.27 (−54.00–86.00) 8.66 ± 52.30 (−60–116)	0.825 ^a^	27.80 ± 54.88 (−76.00–112.00) 2.59 ± 50.50 (−103–100)	0.094 ^a^	0.241 ^c^ 0.749 ^c^
Finger-press reaction time-ND (ms)	1 2	11.53 ± 35.40 (−92.00–57.00) 18.26 ± 64.26 (−36.00–187.00)	0.650 ^b^	−4.33 ± 37.12 (−65.00–70.00) 13.94 ± 32.08 (−58.00–62.00)	0.189 ^a^	0.130 ^c^ 0.245 ^c^
Endurance-D(ms)	1 2	15.17 ± 67.35 (−53.17–170.17) −15.78 ± 38.76 (−112.86–41.95)	0.184 ^a^	−22.55 ± 59.42 (−175.39–72.37) −8.26 ± 54.55 (−103.00–123.97)	0.233^b^	0.272 ^c^ 0.787 ^c^
Endurance-ND (ms)	1 2	3.32 ± 40.55 (−38.50–129.18) 5.41 ± 21.17 (−26.89–57.86)	0.105 ^b^	−25.33 ± 51.83 (−197.94–14.22) 16.76 ± 64.65 (−46.75–176.38)	0.088 ^b^	0.254 ^d^ 0.395 ^d^
Tactile sensitivity-D (g)	1 2	0.12 ± 0.30 (−0.39–0.79) 0.12 ± 0.32 (−0.39–0.78)	0.976 ^a^	0.08 ± 0.34 (−0.47–0.79) −0.08 ± 0.36 (−0.47–0.92)	0.980 ^a^	0.762 ^c^ 0.775 ^c^
Tactile sensitivity-ND (g)	1 2	0.17 ± 0.41 (−0.71–0.79) 0.07 ± 0.30 (−0.39–0.78)	0.471 ^a^	0.08 ± 0.16 (−0.08–0.47) 0.10 ± 0.44 (−0.47–0.93)	0.722 ^b^	0.635 ^d^ 0.850 ^d^
Two-point discrimination-D (mm)	1 2	−0.13 ± 0.83 (−2.00–1.00) 0.06 ± 0.59 (−1.00–1.00)	0.458 ^a^	0.20 ± 0.94 (−1–2) −0.06 ± 0.59 (−1.00–1.03)	0.309 ^a^	0.313^c^ 0.585 ^c^
Two-point discrimination-ND (mm)	1 2	0.26 ± 0.45 (0–1.00) 0.13 ± 0.63 (−1.00–1.00)	0.582 ^a^	0.06 ± 0.70 (−1–1) −0.12 ± 0.64 (−1–1)	0.453 ^a^	0.364 ^c^ 0.367 ^c^
Position sense 125°-D	1 2	−0.33 ± 3.07 (−5.25–6.50) −0.18 ± 4.29 (−5.90–9.65)	0.922 ^a^	−0.96 ± 4.88 (−8.15–8.45) −0.58 ± 4.11 (−9.55–6.55)	0.816 ^a^	0.677 ^c^ 0.798 ^c^
Position sense 125°-ND	1 2	0.04 ± 4.85 (−12.00–9.70) −0.003 ± 3.79 (−8.60–5.80)	1 ^b^	−0.18 ± 4.84 (−9.30–10.50) 0.02 ± 2.99 (−7.15–3.25)	0.889 ^a^	0.468 ^d^ 0.917 ^d^
Position sense 90°-D	1 2	0.003 ± 2.68 (−4.85–4.40) −0.97 ± 2.79 (−8.15–1.80)	0.415 ^a^	−0.09 ± 2.59 (−5.10–4.35) 0.26 ± 2.69 (−3.05–6.95)	0.738 ^a^	0.918 ^c^ 0.228 ^c^
Position sense 90°-ND	1 2	0.58 ± 2.49 (−2.60–5.70) −0.29 ± 3.36 (−6.40–6.15)	0.341 ^a^	0.41 ± 2.89 (−3.55–8.30) −0.24 ± 2.59 (−7.15–3.25)	1 ^b^	0.787 ^c^ 0.803 ^c^
Position sense 55°-D	1 2	0.49 ± 5.21 (−9.15–8.95) 0.18 ± 3.90 (−5.75–9.95)	0.843 ^a^	−0.83 ± 5.43 (−8.85–8.95) −1.13 ± 4.51 (−13.10–5.45)	0.820 ^b^	0.443 ^d^ 0.407 ^d^
Position sense 55°-ND	1 2	−1.50 ± 4.49 (−8.25–5.50) −0.71 ± 4.08 (−10.50–3.65)	0.607 ^a^	−0.53 ± 4.85 (−7.40–7.35) −1.06 ± 2.94 (−5.75–4.50)	0.742 ^a^	0.576 ^c^ 0.789 ^c^
9-Hole Peg-D	1 2	−0.95 ± 2.90 (−4.93–5.29) 0.23 ± 1.80(−3.88–3.54)	0.974 ^b^	−0.92 ± 2.16 (−7.06–1.15) 0.18 ± 1.41(−2.04–2.25)	0.941 ^b^	0.787 ^c^ 0.633 ^c^
9-Hole Peg-ND	1 2	−0.68 ± 1.80(−3.75–2.97) −0.85 ± 1.39(−2.88–2.24)	0.534 ^b^	−1.23 ± 2.86(−7.84–3.58) −0.11 ± 2.68(−4.11–6.17)	0.357 ^b^	0.443 ^c^ 0.407 ^c^

mm: millimeter; ms: milliseconds; g: gram; °: degree; D: dominant; ND: nondominant; ^a^: *t*-test; ^b^: Wilcoxon test; ^c^: independent samples *t*-test; ^d^: Mann–Whitney *U* test.

## Data Availability

The original contributions presented in the study are included in the article.
